# Impact of Location of Residence and Distance to Cancer Centre on Medical Oncology Consultation and Neoadjuvant Chemotherapy for Triple-Negative and HER2-Positive Breast Cancer †

**DOI:** 10.3390/curroncol31080353

**Published:** 2024-08-20

**Authors:** Elliott K. Yee, Julie Hallet, Nicole J. Look Hong, Lena Nguyen, Natalie Coburn, Frances C. Wright, Sonal Gandhi, Katarzyna J. Jerzak, Andrea Eisen, Amanda Roberts

**Affiliations:** 1 Department of Surgery, University of Toronto, Toronto, ON M5S 1A1, Canada; 2 Department of Surgery, Sunnybrook Health Sciences Centre, Toronto, ON M4N 3M5, Canada; 3 Sunnybrook Research Institute, Toronto, ON M4N 3M5, Canada; 4 ICES, Toronto, ON M4N 3M5, Canada; 5 Department of Medicine, University of Toronto, Toronto, ON M5S 1A1, Canada; 6 Department of Medicine, Sunnybrook Health Sciences Centre, Toronto, ON M4N 3M5, Canada

**Keywords:** breast, neoadjuvant, chemotherapy, triple-negative, HER2-positive, geography

## Abstract

Despite consensus guidelines, most patients with early-stage triple-negative (TN) and HER2-positive (HER2+) breast cancer do not see a medical oncologist prior to surgery and do not receive neoadjuvant chemotherapy (NAC). To understand barriers to care, we aimed to characterize the relationship between geography (region of residence and cancer centre proximity) and receipt of a pre-treatment medical oncology consultation and NAC for patients with TN and HER2+ breast cancer. Using linked administrative datasets in Ontario, Canada, we performed a retrospective population-based analysis of women diagnosed with stage I–III TN or HER2+ breast cancer from 2012 to 2020. The outcomes were a pre-treatment medical oncology consultation and the initiation of NAC. We created choropleth maps to assess the distribution of the outcomes and cancer centres across census divisions. To assess the relationship between distance to the nearest cancer centre and outcomes, we performed multivariable regression analyses adjusted for relevant factors, including tumour extent and nodal status. Of 14,647 patients, 29.9% received a pre-treatment medical oncology consultation and 77.7% received NAC. Mapping demonstrated high interregional variability, ranging across census divisions from 12.5% to 64.3% for medical oncology consultation and from 8.8% to 64.3% for NAC. In the full cohort, compared to a distance of ≤5 km from the nearest cancer centre, only 10–25 km was significantly associated with lower odds of NAC (OR 0.83, 95% CI 0.70–0.99). Greater distances were not associated with pre-treatment medical oncology consultation. The interregional variability in medical oncology consultation and NAC for patients with TN and HER2+ breast cancer suggests that regional and/or provider practice patterns underlie discrepancies in the referral for and receipt of NAC. These findings can inform interventions to improve equitable access to NAC for eligible patients.

## 1. Introduction

Breast cancer, defined as malignancy of the breast ducts or lobules, is globally the most commonly diagnosed cancer, and in 2020, caused 685,000 deaths [1]. The mainstay for the treatment of breast cancer has historically been upfront surgery with or without adjuvant (post-operative) chemotherapy, with neoadjuvant (pre-operative) chemotherapy (NAC) used only for locally advanced tumours [2]. However, current evidence-based consensus guidelines recommend the consideration of NAC for most patients with triple-negative (TN) and HER2-positive (HER2+) breast cancers, specifically those with positive lymph nodes (N1-3) and/or tumours measuring 2 cm (cT2) or larger [3,4,5,6]. For these cancers, the clinical response to NAC can guide prognostication and adjuvant therapy, confer axillary lymph node down-staging, and lead to higher rates of breast-conserving surgery [7,8,9,10,11,12,13]. As previously identified by our group in Ontario, Canada, only 1 in 4 women with TN and HER2+ breast cancer are assessed by a medical oncologist and receive NAC prior to surgery despite the majority being eligible for NAC per current recommendations [2]. These findings raise concerns about barriers to accessing care for NAC, when indicated and aligned with patient goals. However, little is known about the mechanisms underlying this discordance with best-practice guidelines.

One potential underlying factor in the use of NAC is geography—both in terms of region of residence and proximity to cancer care—which has been identified as an important mediator of care utilization and can influence survival across a range of surgically treatable cancers [14,15,16,17,18]. In breast cancer care specifically, regional variability in adjuvant chemotherapy and endocrine therapy has been described [19,20]. Greater distance from treating facilities has also been associated with a lower likelihood of receiving adjuvant radiation therapy [21]. However, there is currently no information regarding the potential influence of location of residence and cancer centre proximity on receipt of NAC for TN and HER2+ breast cancer. This study sought to characterize the association between region of residence and cancer centre proximity and the receipt of a pre-treatment medical oncology consultation and NAC for female patients with TN and HER2+ breast cancer in Ontario, Canada, with a view to better understand the receipt of guideline-concordant care and inform efforts to improve it for this patient population.

## 2. Materials and Methods

### 2.1. Study Design and Setting

Using linked administrative healthcare datasets from ICES (formerly known as the Institute for Clinical Evaluative Sciences) in Toronto, ON, Canada, we performed a retrospective population-based cohort study. As of 2016, Ontario’s population of 13,448,494 inhabited a land area of 908,669 km^2^. Per the Canada Health Act, the population under study received universal, publicly funded healthcare through the Ontario Health Insurance Plan (OHIP), allowing for all healthcare encounters during the study period to be captured within the datasets used for this study [22]. The use of the data in this project is authorized under section 45 of Ontario’s Personal Health Information Protection Act (PHIPA) and did not require review by a Research Ethics Board. To protect patient privacy, all data were anonymized and reported in aggregate. This study was reported in accordance with the REporting of studies Conducted using Observational Routinely collected health Data (RECORD) statement [23]. The design of this study was adapted from that of previous geographic studies of pancreatic and esophagogastric cancer within Ontario [24,25].

### 2.2. Study Population and Cohort

The study cohort comprised all patients of the female sex over 18 years old in Ontario with a valid OHIP number and diagnosed with TN or HER2+ breast cancer between 1 April 2012 and 31 January 2020, with stage I to III disease at time of diagnosis, and for whom, at minimum, their estrogen receptor (ER) and HER2 receptor status data were documented. Patients were excluded if no postal code of residence was available (as these patients could not be linked to a location of residence), a second cancer diagnosis occurred within 1 year of the initial breast cancer diagnosis, breast cancer was diagnosed incidentally (defined as a diagnosis of breast cancer on the same date as surgery, as these patients would not have had the opportunity to receive NAC), no treatment with chemotherapy or surgery occurred within 6 months of diagnosis, or no surgery occurred within 12 months of receiving NAC to exclude patients who developed metastases or otherwise developed inoperable disease after NAC. Detailed definitions of the variables used in the creation of the study cohort are described in Table S1.

### 2.3. Data Sources

Detailed information about the data sources is summarized in Tables S1 and S2. These datasets were linked using unique encoded identifiers and analyzed at ICES. Demographic data were obtained from the Ontario Registered Persons Database (RPDB) [26]. Incident breast cancer diagnoses and receptor status (estrogen, progesterone, and HER2) were identified through the Ontario Cancer Registry (OCR). Data about the health services provided were obtained through the OHIP Claims Database, Cancer Activity Level Reporting (ALR), Ontario Drug Benefit (ODB), New Drug Funding Program (NDFP), and Discharge Abstract Database (DAD) [27].

Patient location was determined using the postal code of residence. Ontario comprises 49 geographic administrative regions called census divisions (CDs) [28]. Per the 2016 census, geospatial data defining CDs were obtained from Statistics Canada [29]. Each patient was matched to a unique CD using the Postal Code Conversion File (PCCF), which links postal codes to Canadian census geographic regions [30]. Cancer centres were defined as sites providing chemotherapy according to a contemporary list from Cancer Care Ontario [31]. This comprised all Level 1 (regional cancer centres performing teaching and research), Level 2 (regional cancer centres), Level 3 (affiliate sites), and Level 4 (satellite sites providing chemotherapy without onsite medical oncologists) centres in Ontario, as all provide NAC for breast cancer. The geographic point location of each Level 1–4 cancer centre was defined by latitude and longitude from Google Maps 3.35 (Google, Mountainview, CA, USA), as previously described [24,25].

LN had full access to the databases described above and was responsible for the data linkage, cohort creation, and statistical analyses. EY was responsible for the linkage of aggregated data to the CDs and the creation of choropleth maps.

### 2.4. Exposure

The distance to the nearest cancer centre was the exposure of interest. This was defined as the straight-line distance to the nearest cancer centre from the centroid of a patient’s postal code of residence at the time of diagnosis. Straight-line distance has been applied across numerous geographic studies of cancer care access [18,24,25,32,33,34,35,36,37,38]. Distance was categorized as ≤5 km, 5–10 km, 10–25 km, or ≥25 km to reflect the distance distribution of patients within the cohort.

### 2.5. Outcomes

The outcomes of interest were the receipt of a pre-treatment medical oncology consultation and the initiation of at least one dose of NAC. Pre-treatment was defined as prior to the first treatment (NAC or surgery). Medical oncology consultation was counted if it occurred within 2 months prior to the first treatment. NAC was counted if it occurred within 6 months of diagnosis. A breakdown of the NAC regimens received by the population under study is available in the supplementary materials of Roberts et al., 2024 [2]. The variable definitions and data sources are summarized in Tables S1 and S2.

### 2.6. Covariates

Relevant clinical and sociodemographic covariates were identified a priori based on clinical relevance and the existing literature [24,25]. The included variables were age at diagnosis (categorical), comorbidity burden (continuous), previous breast cancer diagnosis (yes vs. no), first consultation at a regional cancer centre (yes vs. no), deprivation quintile, tumour stage (T0/T1–T4), node stage (N0–N3), and year of diagnosis (2012–2015 vs. 2016–2020), with the latter year category capturing the dissemination of evidence for NAC in TN and HER2+ breast cancer [9,10,39,40]. The comorbidity burden was assigned using the Elixhauser comorbidity index [41,42]. The deprivation quintile is a component of the Ontario Marginalization Index (ON-Marg) and is a geographic areal measure of marginalization assigned based on postal code that captures “income, quality of housing, educational attainment and family structure characteristics” [43,44]. The covariate definitions and data sources are summarized in Tables S1 and S2.

### 2.7. Analysis

Baseline clinical and demographic characteristics were obtained and stratified by distance to the nearest cancer centre. Categorical variables were reported with absolute numbers (*n*) and proportions (%), and the Elixhauser comorbidity index was reported as the mean and standard deviation.

To visualize outcomes across geographic regions, we first constructed choropleth maps with the proportion of medical oncology consultation and the initiation of NAC mapped across CDs in relation to cancer centres for the full cohort. Choropleth maps, which use colour gradients to illustrate outcomes across regions, have previously been used in geographic studies of cancer care [24,25,32]. Patients without CD data were excluded from the mapping analysis. All maps were made with QGIS 2.12 (QGIS Geographic Information System, Open Source Geospatial Foundation Project). Maps were first created for the entire cohort, and subsequently for the two sub-group analyses (diagnosis during 2019–2020, and NAC-eligible).

Next, we assessed the associations between distance to the nearest cancer centre and receipt of a pre-treatment medical oncology consultation and the initiation of NAC, respectively. Multivariable logistic regression models were created and adjusted for the previously mentioned covariates, which were included a priori as potential confounders. Exposure and outcome variables were categorical. All models used generalized estimating equations to account for potential outcome clustering (i.e., non-independence) by the first institution of consultation. The results were reported as the odds ratio (OR) and 95% confidence interval (CI).

A first sub-group analysis restricted to patients diagnosed during 2019–2020 was then conducted, allowing for the uptake of evidence of survival benefit from response-driven adjuvant chemotherapy for TN and HER2+ breast cancer following NAC (specifically, the CREATE-X and KATHERINE trials) [9,10,39,40]. Models were constructed similarly as for the primary analysis, omitting the year of diagnosis as a covariate given that all patients in the sub-cohort fell under the later-year category. A second sub-group analysis was restricted to patients eligible for NAC per the current consensus guidelines (those with stage ≥ cT2 and/or node-positive disease), using the same models as the primary analysis [3,4,5,6].

All multivariable models used a complete case analysis, whereby individuals with missing covariate data were excluded. Data were missing for the deprivation quintile (0.6%), tumour stage (0.2%), and node stage (0.3%). The data were complete for all other covariates. All analyses were two-sided. Statistical significance was defined as *p* < 0.05. All testing was performed with SAS Enterprise Guide 7.1 (SAS Institute Inc., Cary, NC, USA).

## 3. Results

### 3.1. Cohort Description

**Figure 1:**
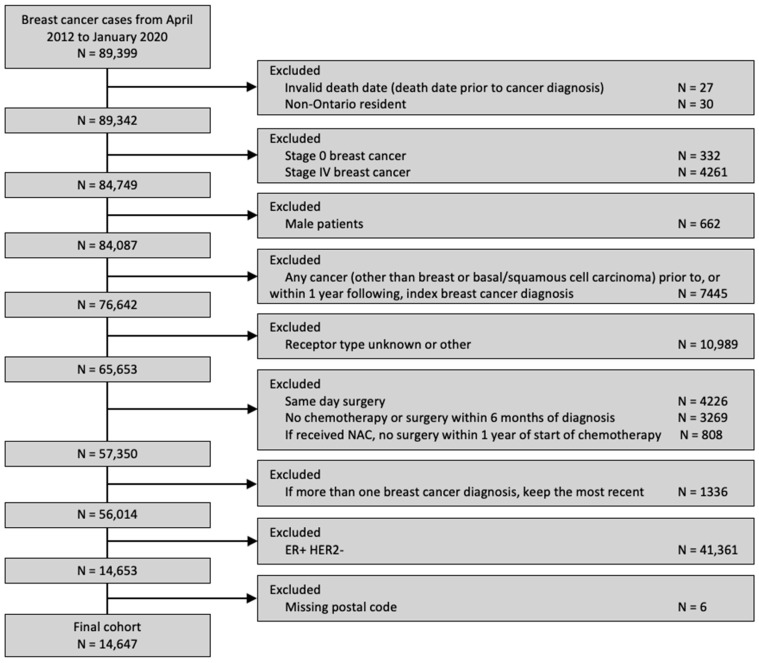
Diagram of cohort creation.

A total of 14,647 patients was included (Figure 1), of whom 29.9% received a pre-treatment medical oncology consultation and 23.9% received NAC. Among the patients who received a pre-treatment medical oncology consultation, 77.7% received NAC. The clinical and sociodemographic information, stratified by distance to the nearest cancer centre, is summarized in Table 1. Most patients (89.5%) resided within 25 km of the nearest cancer centre.

### 3.2. Mapping Analysis

**Figure 2:**
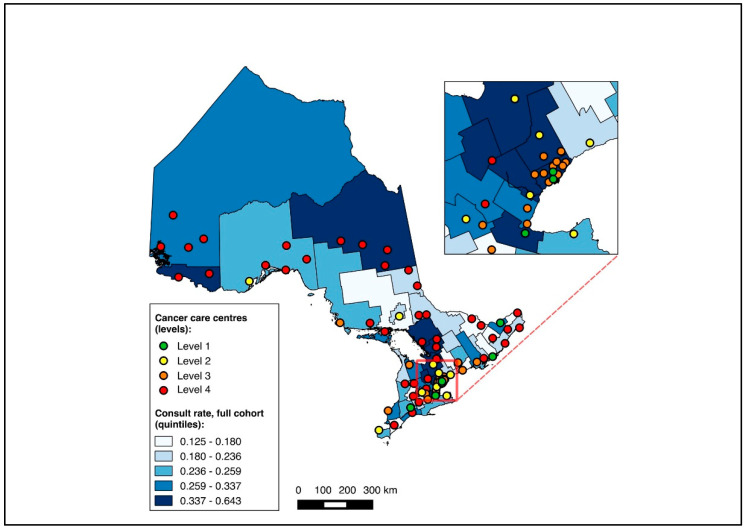
Choropleth map of cancer centres and distribution of pre-treatment medical oncology consultation across census divisions in Ontario for full cohort (2012–2020). Inset map magnifies area with highest cancer centre density.

**Figure 3:**
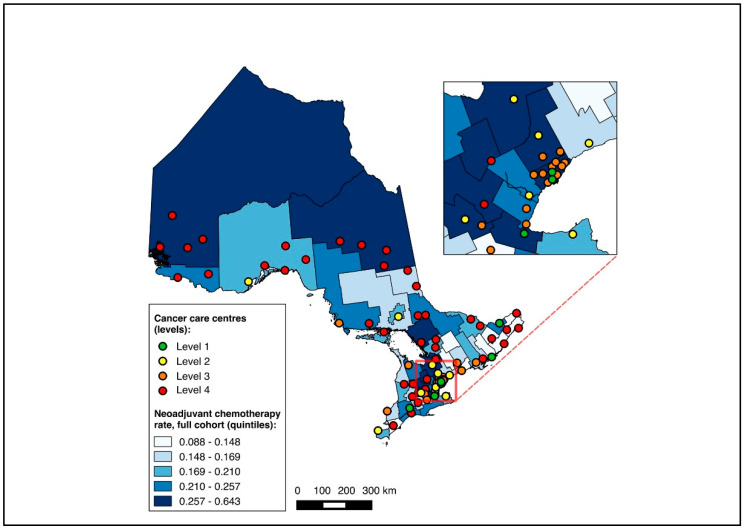
Choropleth map of cancer centres and distribution of neoadjuvant chemotherapy use across census divisions in Ontario for full cohort (2012–2020). Inset map magnifies area with highest cancer centre density.

Overall, 14,643 patients were matched to a CD of residence and included in the choropleth mapping analysis. The choropleth maps of the outcomes by region in relation to the cancer centres for the full cohort (2012–2020) are displayed in Figure 2 and Figure 3, with inset maps magnifying the area with the highest density of cancer centres. The maps demonstrate the high variability in the outcomes across the regions. More densely distributed and higher-level cancer centres predominated in the southern regions of the province, in contrast to sparser and lower-level cancer centres in the northern regions. Pre-treatment medical oncology consultation ranged from 12.5% to 64.3% (Figure 2), and NAC from 8.8% to 64.3% (Figure 3) across CDs. The CDs with the highest proportions of pre-treatment medical oncology consultation and NAC were distributed across both the southern and northern regions of the province.

### 3.3. Association between Distance and Outcomes for Full Cohort

**Figure 4:**
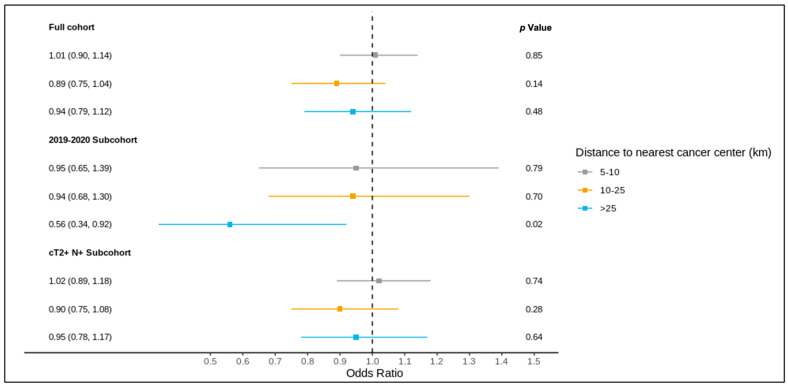
Results of multivariable logistic regression of the association between distance to the nearest cancer centre and receipt of pre-treatment medical oncology consultation for the full cohort, 2019–2020 sub-cohort, and NAC-eligible (≥cT2 and/or node-positive) sub-cohort, reported as the odds ratio (95% confidence interval) and *p* value, and adjusted for age, comorbidity burden, previous breast cancer diagnosis, first consultation at regional cancer centre, deprivation, year of diagnosis (except for the 2019–2020 sub-cohort), tumour stage, and node stage.

**Figure 5:**
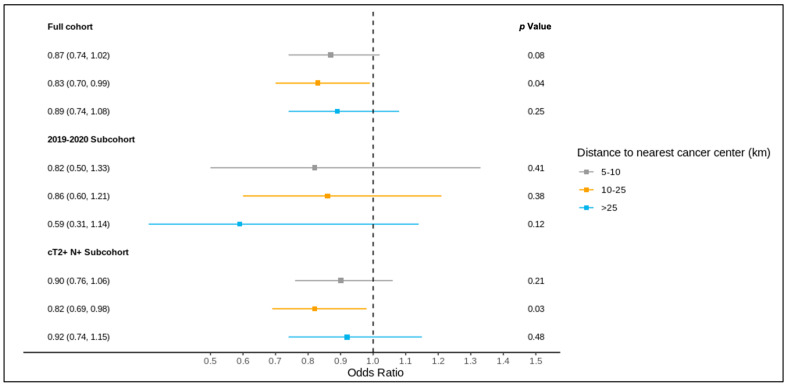
Results of multivariable logistic regression of the association between distance to the nearest cancer centre and receipt of NAC for the full cohort, 2019–2020 sub-cohort, and NAC-eligible (≥cT2 and/or node-positive) sub-cohort, reported as the odds ratio (95% confidence interval) and *p* value, and adjusted for age, comorbidity burden, previous breast cancer diagnosis, first consultation at regional cancer centre, deprivation, year of diagnosis (except for the 2019–2020 sub-cohort), tumour stage, and node stage.

The results of the full cohort multivariable logistic regression analysis are summarized in Figure 4 and Figure 5 and Table S3. Compared to patients living ≤5 km from the nearest cancer centre, residence at a greater distance (5–10 km, 10–25 km, >25 km) was not associated with different odds of receiving a pre-treatment medical oncology consultation. A distance of 10–25 km from the nearest cancer centre was, however, significantly associated with lower odds of receiving NAC (adjusted OR 0.83, 95% CI 0.70–0.99) compared to ≤5 km.

### 3.4. Sub-Group Analysis: Diagnosis over 2019–2020

**Figure 6:**
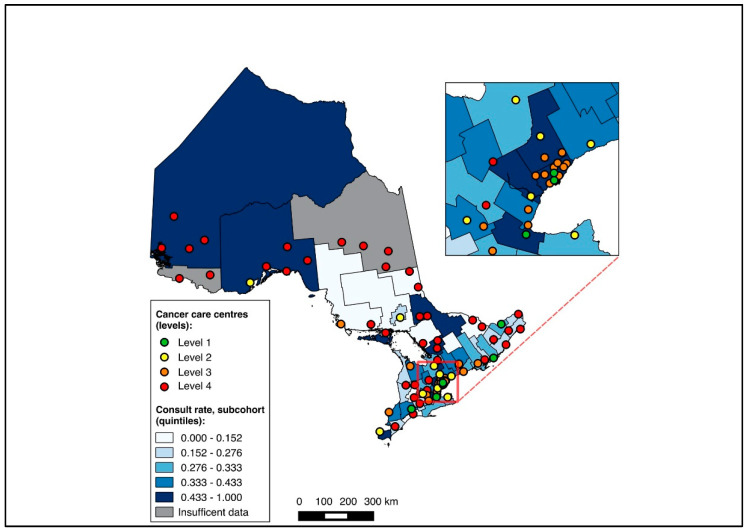
Choropleth map of cancer centres and distribution of pre-treatment medical oncology consultation across census divisions in Ontario for the 2019–2020 sub-cohort. The inset map magnifies the area with the highest cancer centre density.

**Figure 7:**
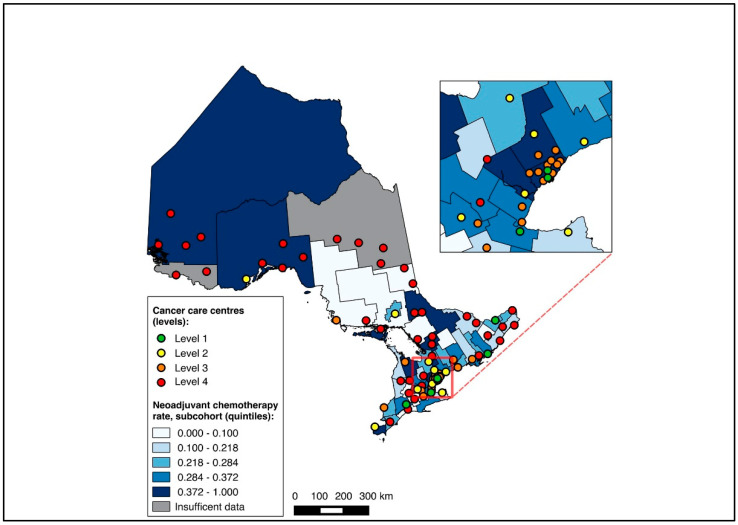
Choropleth map of cancer centres and distribution of neoadjuvant chemotherapy use across census divisions in Ontario for the 2019–2020 sub-cohort. The inset map magnifies the area with the highest cancer centre density.

Of all patients, 1621 were diagnosed over 2019–2020 and included in this sub-group analysis, of whom 1619 were matched to a CD of residence for the mapping analysis. A pre-treatment medical oncology consultation was received by 40.8% of this sub-group, and NAC by 32.5%, with 78.2% of those receiving a pre-treatment medical oncology consultation also receiving NAC (in contrast, among the patients diagnosed over 2012–2018, 28.6% received a pre-treatment medical oncology consultation, 22.8% received NAC, and 77.6% of those who received a pre-treatment medical oncology consultation received NAC). Across the CDs, the rates of pre-treatment medical oncology consultation and NAC both ranged from 0.0% to 100.0% for the 2019–2020 sub-group (Figure 6 and Figure 7). The outcomes were geographically distributed throughout the province, similarly to the primary mapping analysis. A distance of >25 km from the nearest cancer centre was significantly associated with lower odds of a pre-treatment medical oncology consultation (adjusted OR 0.56, 95% CI 0.34–0.92) compared to ≤5 km (Figure 4, Table S4). Greater distance was not significantly associated with receiving NAC (Figure 5, Table S4).

### 3.5. Sub-Group Analysis: NAC-Eligible

**Figure 8:**
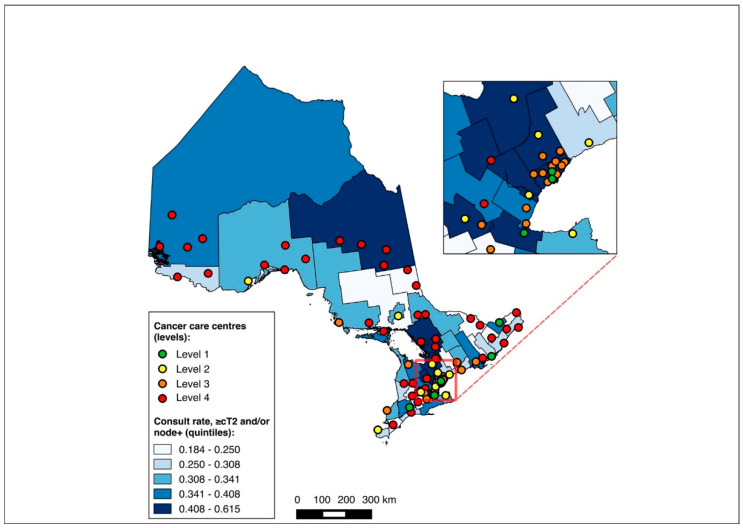
Choropleth map of cancer centres and distribution of pre-treatment medical oncology consultation across census divisions in Ontario for the ≥cT2 and/or node-positive sub-cohorts. The inset map magnifies the area with the highest cancer centre density.

**Figure 9:**
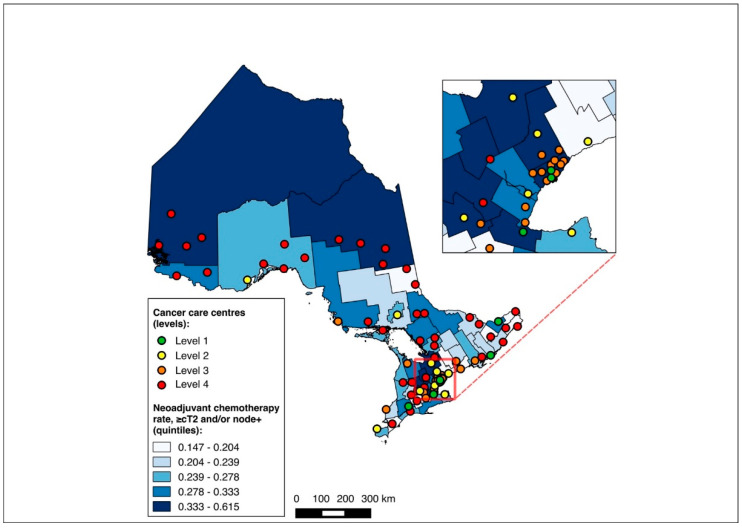
Choropleth map of cancer centres and distribution of neoadjuvant chemotherapy use across census divisions in Ontario for the ≥cT2 and/or node-positive sub-cohorts. The inset map magnifies the area with the highest cancer centre density.

The NAC-eligible sub-group (≥cT2 and/or node-positive disease) included 10,055 patients (68.6% of the study cohort), of whom all were matched to a CD of residence for the mapping analysis. A pre-treatment medical oncology consultation was received by 38.6% of this sub-group, and NAC by 32.4%, with 82.0% of those who received a pre-treatment medical oncology consultation also receiving NAC. Across the CDs, the rate of pre-treatment medical oncology consultation ranged from 18.4% to 61.5% (Figure 8), and that of NAC from 14.7% to 61.5% (Figure 9). The outcomes were geographically distributed throughout the province, similarly to the primary mapping analysis. As with the primary multivariable analysis, a distance of 10–25 km from the nearest cancer centre was significantly associated with lower odds of receiving NAC (adjusted OR 0.82, 95% CI 0.69–0.98) compared to ≤5 km, whereas greater distance was not significantly associated with receiving a pre-treatment medical oncology consultation (Figure 4 and Figure 5, Table S5).

## 4. Discussion

In this population-based cohort study, we assessed the association between distance to the nearest cancer centre and receipt of pre-treatment medical oncology consultation and NAC for women with TN and HER2+ breast cancer, as well as the geographical distribution of receipt of care across a healthcare system. Within our cohort, a minority of women received a pre-treatment medical oncology consultation and NAC, which remained true even for the sub-group diagnosed over 2019–2020 and those with NAC-eligible disease per the current guidelines. Across the main cohort and sub-groups, the mapping analysis demonstrated wide variability in the outcomes across the regions. Multivariable analyses, while identifying an association between 10–25 km and lower odds of NAC (and lower odds of pre-treatment medical oncology consultation at >25 km for the 2019–2020 sub-group), it did not demonstrate any consistent distance–outcome relationship across the main cohort and sub-groups. Neoadjuvant treatment for TN and HER2+ breast cancer has evidently changed over time, with rates of pre-treatment medical oncology consultation increasing from 28.6% to 40.8% and those of NAC from 22.8% to 32.5% between 2012–2018 and 2019–2020. This is temporally consistent with the initial presentation and publication of the CREATE-X and KATHERINE trials [9,10,39,40]. Although these upward trends likely reflect the uptake of evidence into clinical practice, discordance between the guidelines and the real-world treatment patterns persist, with high variability in pre-treatment medical oncology consultation and NAC receipt across CDs in the 2019–2020 sub-group analysis. These findings indicate region-level opportunities to improve the receipt of pre-treatment medical oncology consultation and NAC for women with TN and HER2+ breast cancer, and they contribute to a growing body of literature on the influence of geography in breast cancer care systems.

Regional variability has been described for the receipt of chemotherapy, surgery, radiation therapy, surveillance, and treatment monitoring, as well as cost, adherence to practice guidelines, and survival in breast cancer care [19,20,32,45,46,47,48,49,50,51,52,53,54,55,56]. Proximity to cancer care has also been associated with diagnosis and outcomes for breast cancer, with greater travel times associated with a higher stage at diagnosis and worse survival, and greater distance from cancer centres associated with a lower likelihood of receiving adjuvant radiation therapy [21,57]. By examining the influence of both region of residence and proximity to cancer care within a large and geographically diverse population-based cohort, our study provides novel information specific to the care of patients with TN and HER2+ breast cancer. Nearly a decade after the first presentation of landmark trials demonstrating survival benefit from response-driven adjuvant chemotherapy for TN and HER2+ breast cancer following NAC (CREATE-X and KATHERINE), our analysis was needed to explore factors underlying the slow uptake of NAC in practice (particularly important now, given the upholding of the KATHERINE trial’s initial findings and demonstration of improved overall survival in its most recent 2023 update) [9,10,39,40,58]. Consistent with a prior report from our group on this cohort, a minority of patients with TN and HER2+ breast cancer received a pre-treatment medical oncology consultation (29.9%) and NAC (23.9%) [2]. While this was slightly improved within the NAC-eligible (≥cT2 and/or node-positive) sub-group, receipt of pre-treatment medical oncology consultation remained limited to approximately 2 out of 5 patients, and NAC to approximately 1 out of 3 patients. Our findings indicate that some elements of geography—specifically the region of residence, and to a lesser extent, distance to the nearest cancer centre—may influence the receipt of care for this patient population.

Our study shows important findings regarding the location of one’s residence in relation to cancer centres. First, we observed a non-linear dose–response relationship between distance to the nearest cancer centre and the receipt of NAC in our primary analysis. Residing at a moderate distance from the nearest cancer centre (10–25 km in this analysis) was associated with lower odds of receiving NAC compared to ≤5 km, yet this association was not seen at greater distances. This non-linear relationship could be influenced by mitigating individual and system factors at more extreme distances [24,25,33,59,60,61,62,63]. Similar trends in oncology consultation and receipt of systemic therapy within Ontario, in relation to distance and region, have previously been reported for pancreatic and esophagogastric cancer [24,25]. Second, distance to the nearest cancer centre was not associated with the receipt of a pre-treatment medical oncology consultation in our primary analysis. With a large sample size and narrow confidence intervals, we do not believe that this reflects an issue with statistical power. Rather, this finding points toward differences in decision-making after patients see medical oncology. While distance does not appear to significantly influence the receipt of consultation, these findings suggest that once seen by medical oncology, some patients are less likely than others to receive NAC. This difference in decision-making could be at the level of the medical oncologist, the multidisciplinary team, or the patient, which cannot be ascertained within our study design. For instance, the intermediate distance category (10–25 km) may reflect patients living in distinct communities with different practice settings and lower guideline concordance, different care network organization (such as access to or use of multidisciplinary tumour board review), or different social supports and patient preferences. Third, beyond distance to cancer centres, our mapping analysis demonstrated marked variability in the receipt of pre-treatment medical oncology consultation and NAC across CDs, indicating potential inequalities in care. Despite greater concentrations (and higher levels) of cancer centres in the southern CDs, CDs with high and low receipt of consultation and NAC were distributed throughout both the northern and southern regions of the province. These findings suggest that the community of residence, and not merely the distance to the nearest cancer centre, may be driving the difference in utilization of NAC seen at a moderate distance from cancer centres. The reasons underlying these differences, and whether they influence outcomes such as survival, fell beyond the scope of this study but will be addressed in future work. These three findings were consistent in the primary analysis and the sub-group of NAC-eligible patients.

Our study findings bear implications for future research and for the design of strategies to increase the uptake of NAC for TN and HER2+ breast cancer. Understanding and addressing geography-related variability in referral pathways and NAC for TN and HER2+ breast cancer—for which local referral pathways, clinician knowledge and practice patterns, and resource availability may be underlying mechanisms—should be prioritized to improve the design of cancer systems. Our group has previously observed that the vast majority of patients with TN and HER2+ breast cancer are seen first by a surgeon following diagnosis, highlighting the surgeon–medical oncologist referral pathway as an important target for further study [2]. Within these referral pathways, attention should be paid to clinicians in geographic regions with low NAC guideline concordance, as they appear to represent a source of unrealized treatment potential. Such provider-level differences across geographic contexts are a well-described phenomenon. Among surgeons, regional variability in clinical decision making, while multifactorial, may be influenced by individual beliefs and preferences [64]. Similarly, medical oncologists’ practice structures may vary significantly between rural and urban locations [65]. Thus, efforts are warranted to better understand why certain regions exhibit low or high NAC guideline concordance, which requires further study of provider knowledge and preferences. In settings where knowledge gaps may exist, cancer care providers have previously indicated a need for greater availability of accessible clinical practice guideline summaries, highlighting one possible avenue to improve guideline concordance [66]. Our findings also provide opportunities for clinicians practicing in regions with low medical oncology referrals and NAC receipt to consider how their local referral pathways may be optimized to reduce disparities in care access. Finally, region-specific resource limitations are known to influence clinician decision-making in referring to specialist cancer care, and may pose an additional barrier to oncology referral and receipt of NAC [67,68]. Thus, ensuring local access to medical oncologists and cancer centres is crucial in the design of regional cancer systems. Where resource limitations persist, virtual cancer care—while not measured within this study—has seen wide uptake during the COVID-19 pandemic and may represent an important strategy for overcoming geographic barriers in oncology consultation and NAC coordination [69,70,71,72]. Nevertheless, given that the receipt of NAC itself is not amenable to virtual care, any integration of telemedicine into existing referral systems should occur alongside efforts to improve access to physical cancer care infrastructure.

This study has limitations. We used data routinely collected for administrative purposes and not our specific research question. As such, some information was not available, such as individual provider- and patient-level data pertaining to care decisions. Thus, we could not adjust for potential confounding from such unmeasured factors that might be associated with both patient distance from cancer centres and tendency to seek or receive care. However, given that greater distances have been described in the cancer literature as both deleterious and protective toward the receipt of care and clinical outcomes, and given that we did not observe a consistent distance–outcome relationship within our cohort, it is unlikely that such confounding would substantially influence our results [24,25,33,59,60]. We were also unable to account for care obtained outside of Ontario and did not measure changes in patient residence during the study period, although such events are typically rare. The findings of our choropleth mapping analysis are ecologic, limiting their direct applicability to specific individuals within geographic areas. Such region-based analyses may also be biased by the often arbitrary definitions of jurisdictional boundaries [73,74,75]. Owing to the large number of CDs and relatively few events within certain CDs, we did not perform statistical analysis comparing outcomes across regions, limiting quantitative inference from these maps alone. Rather, the mapping of outcomes serves to visualize geographic trends, generate regional hypotheses, and supplement our multivariable distance analysis. Additionally, since data collection was limited to diagnoses up to January 2020, we cannot comment on long-term changes in healthcare delivery related to the COVID-19 pandemic, such as the uptake of virtual cancer care or the avoidance of in-person care, such as NAC. Although outside the scope of this study, future research should consider how these system-level changes may influence current care delivery for TN and HER2+ breast cancer. Despite these limitations, this study has unique strengths. The large size of the cohort and area under study allowed us to investigate a range of geographic contexts, strengthening the generalizability of findings across geographically diverse settings. Additionally, as this study was conducted in a universal healthcare system, receipt of care was not confounded by insurance status. Regional variability in medical oncology care for breast cancer has nevertheless also been described in the United States, suggesting that the mechanisms driving our observed regional care disparities are relevant in jurisdictions with different healthcare funding models [19,20].

## 5. Conclusions

Despite high-level evidence and consensus guidelines, most women with TN and HER2+ breast cancer do not receive a pre-treatment medical oncology consultation or NAC. Although residing at moderate distances from cancer centres appears to influence the receipt of NAC, this distance–care relationship is not linear, and interregional variability in both pre-treatment medical oncology consultation and receipt of NAC is evident. This geographic variability may reflect access to care and practice differences across geographic regions and within specific communities. Further attention is needed to elucidate local and provider-specific referral and treatment patterns, which can be leveraged to increase the uptake of NAC and improve outcomes for patients with TN and HER2+ breast cancer. The surgery–medical oncology referral pathway, access to evidence-based practice guidelines, and region-specific resource constraints are potential areas for interventions to reduce variations in NAC receipt across regions.

## Figures and Tables

**Table 1:** Characteristics of cohort, stratified by distance to nearest Level 1–4 cancer centre. * Exact number suppressed to prevent small cell identification.

Characteristic		≤5 km	5–10 km	10–25 km	>25 km	All Patients	*p* Value
Patients—*n*		6081	3588	3436	1542	14,647	
Year of diagnosis—*n* (%)	2012–2015	2862 (47.1)	1696 (47.3)	1540 (44.8)	749 (48.6)	6847 (46.7)	0.05
	2016–2020	3219 (52.9)	1892 (52.7)	1896 (55.2)	793 (51.4)	7800 (53.3)	
Age, years—*n* (%)	≤40	607 (10.0)	355 (9.9)	324 (9.4)	104 (6.7)	1390 (9.5)	<0.0001
	41–50	1244 (20.5)	755 (21.0)	677 (19.7)	203 (13.2)	2879 (19.7)	
	51–60	1605 (26.4)	1017 (28.3)	1004 (29.2)	452 (29.3)	4078 (27.8)	
	61–70	1330 (21.9)	804 (22.4)	846 (24.6)	429 (27.8)	3409 (23.3)	
	71–80	864 (14.2)	470 (13.1)	423 (12.3)	257 (16.7)	2014 (13.8)	
	>81	431 (7.1)	187 (5.2)	162 (4.7)	97 (6.3)	877 (6.0)	
Deprivation quintile—*n* (%)	1st (least deprived)	1083 (17.8)	985 (27.5)	1008 (29.3)	245 (15.9)	3321 (22.7)	<0.0001
	2nd	1001 (16.5)	899 (25.1)	831 (24.2)	339 (22.0)	3070 (21.0)	
	3rd	1067 (17.5)	687 (19.1)	765 (22.3)	390 (25.3)	2909 (19.9)	
	4th	1240 (20.4)	593 (16.5)	568 (16.5)	311 (20.2)	2712 (18.5)	
	5th (most deprived)	1674 (27.5)	414 (11.5)	240 (7.0)	225 (14.6)	2553 (17.4)	
	Unknown	16 (0.3)	10 (0.3)	24 (0.7)	32 (2.1)	82 (0.6)	
Elixhauser comorbidity score—mean (standard deviation)		0.14 (0.57)	0.11 (0.49)	0.11 (0.49)	0.14 (0.54)	0.13 (0.53)	<0.01
Previous breast cancer—*n* (%)	No	5556 (91.4)	3298 (91.9)	3169 (92.2)	1405 (91.1)	13,428 (91.7)	0.38
	Yes	525 (8.6)	290 (8.1)	267 (7.8)	137 (8.9)	1219 (8.3)	
Tumour stage—*n* (%)	T0/T1	2373 (39.0)	1428 (39.8)	1357 (39.5)	670 (43.5)	5828 (39.8)	<0.01
	T2	2784 (45.8)	1618 (45.1)	1548 (45.1)	678 (44.0)	6628 (45.3)	
	T3	552 (9.1)	348 (9.7)	350 (10.2)	110 (7.1)	1360 (9.3)	
	T4	365 (6.0)	189–193 *	170 (4.9)	79–83 *	804 (5.5)	
	Unknown	7 (0.1)	≤5 *	11 (0.3)	≤5*	27 (0.2)	
Node stage—*n* (%)	N0	3583 (58.9)	2115 (58.9)	2054 (59.8)	947 (61.4)	8699 (59.4)	0.45
	N1	1889 (31.1)	1132 (31.5)	1069 (31.1)	460 (29.8)	4550 (31.1)	
	N2	384 (6.3)	215 (6.0)	199 (5.8)	89 (5.8)	887 (6.1)	
	N3	214 (3.5)	112 (3.1)	100–104 *	41–45 *	471 (3.2)	
	Unknown	11 (0.2)	14 (0.4)	10–14 *	≤5 *	40 (0.3)	
First consultation at regional cancer centre—*n* (%)	No	3081 (50.7)	1778 (49.6)	1900 (55.3)	909 (58.9)	7668 (52.4)	<0.0001
	Yes	3000 (49.3)	1810 (50.4)	1536 (44.7)	633 (41.1)	6979 (47.6)	

## Data Availability

The dataset from this study is held securely in coded form at ICES. While legal data sharing agreements between ICES and data providers (e.g., healthcare organizations and the government) prohibit ICES from making the dataset publicly available, access may be granted to those who meet pre-specified criteria for confidential access, available at www.ices.on.ca/DAS (accessed on 13 August 2024) (email: das@ices.on.ca). The full dataset creation plan and underlying analytic code are available from the authors upon request, with the understanding that the computer programs may rely upon coding templates or macros that are unique to ICES and are, therefore, either inaccessible or may require modification (source: “ICES Manuscript Checklist, 15 November 2022”).

## Supplementary Materials

The following supporting information can be downloaded at: https://www.mdpi.com/article/10.3390/curroncol31080353/s1, Table S1. Variable definitions. Table S2. Data sources. Table S3. Adjusted odds ratios for association between distance to nearest cancer centre and outcomes in full cohort (2012–2020). Table S4. Adjusted odds ratios for association between distance to nearest cancer centre and outcomes in 2019–2020 subcohort. Table S5. Adjusted odds ratios for association between distance to nearest cancer centre and outcomes in ≥cT2 and/or node-positive subcohort.

## Abbreviations

ALR—Activity Level Reporting, DAD—Discharge Abstract Database, NDFP—New Drug Funding Program, OCR—Ontario Cancer Registry, ODB—Ontario Drug Benefit, OHIP—Ontario Health Insurance Plan, ON-Marg—Ontario Marginalization Index, RPDB—Registered Persons Database.
