# Supplementary material for: Impact of Location of Residence and Distance to Cancer Centre on Medical Oncology Consultation and Neoadjuvant Chemotherapy for Triple-Negative and HER2-Positive Breast Cancer
Source: Curr Oncol. 2024 Aug 20;31(8):4728–45. doi: 10.3390/curroncol31080353 (PMC11352802; doi:10.3390/curroncol31080353)
Supplement: Supplementary file 1 [file curroncol-31-00353-s001.zip › curroncol-3114195-supplementary.pdf]

Supplementary Materials

Table S1. Variable definitions.

Variable	Data source	Code strategy
Breast cancer diagnosis	OCR	Patients in OCR with ICD-10 breast cancer diagnosis code C50x
Receptor status	OCR	Prior to 2018, Collaborative Stage Site-Specific Factor (CS SSF) was used to identify ER status (CS SSF 1), PR status (CS SSF 2), and HER2 status (CS SSF 15). After 2018, OCR variables ER_SUMMARY, PR_SUMMARY, and HER2_OVERALL_SUMMARY were used.
Surgery	OHIP	OHIP fee codes R107, R111, R108, R109, R117, E546, R913, R914, Z427
Same day surgery	OHIP	Breast cancer surgery on same day as breast cancer diagnosis
Death	RPDB	Date of death
T stage	OCR	---
N stage	OCR	---
Medical oncology consultation	OHIP	OHIP fee codes A445, A135, A615, A130, A446, A435, A655, A845, A136, A616, A443, A133, A613, A441, A131, A611, A444, A134, A614, A448, A138, A618, C445, C845, C446, C443, C444 (within 2 months prior to first treatment)
Neoadjuvant chemotherapy	ODB, ALR, NDFP	Receipt of neoadjuvant chemotherapy drugs by intravenous administration in ODB, ALR, or NDFP (within 6 months of diagnosis)
Age at diagnosis	RPDB	---
Deprivation	ON-Marg	Calculated based on postal code of residence using the Ontario Marginalization Index[1,2]

Elixhauser comorbidity index	DAD	Calculated based on hospitalizations within 2 years prior to index breast cancer diagnosis using the Elixhauser index[3,4,5]
First consultation at a Regional Cancer Centre	OHIP	First consultation performed by a physician who also billed at an RCC within ±2 years of consultation date

Abbreviations: ALR – Activity Level Reporting, DAD – Discharge Abstract Database, NDFP – New Drug Funding Program, OCR – Ontario Cancer Registry, ODB – Ontario Drug Benefit, OHIP – Ontario Health Insurance Plan, ON-Marg – Ontario Marginalization Index, RPDB – Registered Persons Database

References:

1. Matheson FI, Moloney G, van Ingen T: 2016 Ontario Marginalization Index User Guide, 2018
2. Matheson FI, Dunn JR, Smith KL, et al: Development of the Canadian Marginalization Index: A New Tool for the Study of Inequality. Canadian Journal of Public Health 103:S12-16, 2012
3. Elixhauser A, Steiner C, Harris DR, et al: Comorbidity Measures for Use with Administrative Data. Medical Care 36:8–27, 1998
4. Gutacker N, Bloor K, Cookson R: Comparing the performance of the Charlson/Deyo and Elixhauser comorbidity measures across five European countries and three conditions. European Journal of Public Health 25:15–20, 2015
5. Juurlink D, Preyra C, Croxford R, et al: Canadian Institute for Health Information Discharge Abstract Database: A Validation Study. Toronto, Institute for Clinical Evaluative Sciences, 2006

Table S2. Data sources.

Database	Description
Activity Level Reporting (ALR)	The ALR contains information at the patient level about services provided within the cancer care system, including radiation therapy, systemic therapy, and outpatient clinic visits.
Discharge Abstract Database (DAD)	The DAD contains information from hospital discharges, including diagnoses and comorbidities.
New Drug Funding Program (NDFP)	The NDFP is administered by Cancer Care Ontario and provides funding for injectable cancer drugs.
Ontario Cancer Registry (OCR)	The OCR is a provincial registry that contains information about all incident breast cancer diagnoses in Ontario.
Ontario Drug Benefit (ODB)	The ODB provides prescription drug coverage for patients ≥65 years old (and certain patients <25 years old since 2018).
Ontario Health Insurance Plan (OHIP) Claims Database	The OHIP Claims Database contains information on physician services billed under Ontario's publicly funded, universal healthcare system.
Ontario Marginalization Index (ON-Marg)	The ON-Marg contains area-based information about multiple measures of marginalization across a variety of geographic levels in Ontario using census data.
Registered Persons Database (RPDB)	The RPDB contains demographic information about individuals in Ontario who have received a health card.

Table S3. Adjusted odds ratios for association between distance to nearest cancer centre and outcomes in full cohort (2012-2020).

	Pre-treatment medical oncology consultation (full cohort)			Neoadjuvant chemotherapy (full cohort)		
Distance to nearest cancer centre (km)	Odds ratio*	95% CI	p-value	Odds ratio*	95% CI	p-value
≤5 (reference)						
5-10	1.01	0.90-1.14	0.851	0.87	0.74-1.02	0.077
10-25	0.89	0.75-1.04	0.143	0.83	0.70-0.99	0.037
>25	0.94	0.79-1.12	0.479	0.89	0.74-1.08	0.254

*Adjusted for age, comorbidity burden, previous breast cancer diagnosis, first consultation at regional cancer centre, deprivation, tumour stage, node stage, year of diagnosis

Table S4. Adjusted odds ratios for association between distance to nearest cancer centre and outcomes in 2019-2020 subcohort.

	Pre-treatment medical oncology consultation (subcohort)			Neoadjuvant chemotherapy (subcohort)		
**Distance to nearest cancer centre (km)**	**Odds ratio***	**95% CI**	**p-value**	**Odds ratio***	**95% CI**	**p-value**
≤5 (reference)						
5-10	0.95	0.65-1.39	0.790	0.82	0.50-1.33	0.411
10-25	0.94	0.68-1.30	0.695	0.86	0.60-1.21	0.382
>25	0.56	0.34-0.92	0.022	0.59	0.31-1.14	0.119

*Adjusted for age, comorbidity burden, previous breast cancer diagnosis, first consultation at regional cancer centre, deprivation, tumour stage, node stage

Table S5. Adjusted odds ratios for association between distance to nearest cancer centre and outcomes in ≥cT2 and/or node-positive subcohort.

	Pre-treatment medical oncology consultation (subcohort)			Neoadjuvant chemotherapy (subcohort)		
**Distance to nearest cancer centre (km)**	**Odds ratio***	**95% CI**	**p-value**	**Odds ratio***	**95% CI**	**p-value**
≤5 (reference)						
5-10	1.02	0.89-1.18	0.739	0.90	0.76-1.06	0.211
10-25	0.90	0.75-1.08	0.275	0.82	0.69-0.98	0.031
>25	0.95	0.78-1.17	0.644	0.92	0.74-1.15	0.479

*Adjusted for age, comorbidity burden, previous breast cancer diagnosis, first consultation at regional cancer centre, deprivation, tumour stage, node stage, year of diagnosis
